# The Ocular Neural Crest: Specification, Migration, and Then What?

**DOI:** 10.3389/fcell.2020.595896

**Published:** 2020-12-23

**Authors:** Antionette L. Williams, Brenda L. Bohnsack

**Affiliations:** ^1^Division of Ophthalmology, Ann & Robert H. Lurie Children’s Hospital of Chicago, Chicago, IL, United States; ^2^Department of Ophthalmology, Northwestern University Feinberg School of Medicine, Chicago, IL, United States

**Keywords:** neural crest, periocular mesenchyme, ocular development, anterior segment, ocular diseases

## Abstract

During vertebrate embryonic development, a population of dorsal neural tube-derived stem cells, termed the neural crest (NC), undergo a series of morphogenetic changes and extensive migration to become a diverse array of cell types. Around the developing eye, this multipotent ocular NC cell population, called the periocular mesenchyme (POM), comprises migratory mesenchymal cells that eventually give rise to many of the elements in the anterior of the eye, such as the cornea, sclera, trabecular meshwork, and iris. Molecular cell biology and genetic analyses of congenital eye diseases have provided important information on the regulation of NC contributions to this area of the eye. Nevertheless, a complete understanding of the NC as a contributor to ocular development remains elusive. In addition, positional information during ocular NC migration and the molecular pathways that regulate end tissue differentiation have yet to be fully elucidated. Further, the clinical challenges of ocular diseases, such as Axenfeld-Rieger syndrome (ARS), Peters anomaly (PA) and primary congenital glaucoma (PCG), strongly suggest the need for better treatments. While several aspects of NC evolution have recently been reviewed, this discussion will consolidate the most recent current knowledge on the specification, migration, and contributions of the NC to ocular development, highlighting the anterior segment and the knowledge obtained from the clinical manifestations of its associated diseases. Ultimately, this knowledge can inform translational discoveries with potential for sorely needed regenerative therapies.

## Introduction

The neural crest (NC) is an embryonic population of multipotent cells that are extremely important for vertebrate body development. Concomitant with gastrulation, induction at the neural plate border leads to the delamination of cells from the neural ectoderm during primary neurulation (Williams and Bohnsack, 2015, 2019; Gandhi and Bronner, 2018; Pla and Monsoro-Burq, 2018; Rogers and Nie, 2018; Szabo and Mayor, 2018; Prasad et al., 2019). These early NC cells then undergo an epithelial-to-mesenchymal transition (EMT) and subsequently migrate throughout the body to form a diverse set of cells and tissues, including bone, cartilage, peripheral neurons, and melanocytes. NC cells derived from the prosencephalon, mesencephalon, and rhombencephalon comprise the cranial subpopulation that contributes to the frontonasal process, periocular mesenchyme (POM) and pharyngeal arches. Through these embryonic structures and cell populations, NC cells then give rise to craniofacial connective, skeletal and neuronal tissues (Cordero et al., 2011; Kish et al., 2011). Furthermore, cranial NC cells within the POM enter the anterior segment of the eye and form parts of the cornea, iris, sclera, ciliary body, and aqueous outflow pathways (Whikehart, 2010; Cordero et al., 2011; Kish et al., 2011; Williams and Bohnsack, 2015).

As an extremely dynamic cell population, the NC is of high clinical interest, as disruption of this cell population results in a wide range of congenital abnormalities (Bohnsack and Kahana, 2013; Williams and Bohnsack, 2015, 2019; Akula et al., 2019). Since described by Wilheim His in 1868, our understanding of NC biology has grown, and the unique characteristics of NC cells have been an interesting and well-studied topic. Comparative analyses across multiple diverse vertebrate model organisms have elucidated the progressive and conserved steps involved in NC development from induction to cell specification, delamination, emigration, and eventual differentiation (Simoes-Costa and Bronner, 2013; Green et al., 2015). Diligent and systematic work in this field has revealed a host of regulatory genes and interactions to further tease out the gene regulatory network underlying complex NC development (Simoes-Costa and Bronner, 2015; Martik and Bronner, 2017). However, much of the evidence pertains to the genetic networks responsible for ectodermal and neurectodermal patterning, and little is known about the specification of the ocular NC. Indeed, the continued requirement of NC stem cells for the maintenance of adult eyes (Jinno et al., 2010; Green et al., 2015; Chawla et al., 2018) emphasizes the importance of this cell population over the vertebrate lifetime. This review will provide an update of the morphogenetic, gene regulation and signaling events controlling ocular NC migration and differentiation, with particular emphasis on the ocular anterior segment. The current challenge is to determine the molecular events that accompany distinct positional stages, as fate maps of the various ocular NC populations are lacking, and there is little to no information on the biology underlying end tissue differentiation in the eye. An adequate understanding of ocular evolution, particularly anterior segment development, and the pathogenesis of its associated disorders will bring us closer to deciphering the mechanisms that participate in the normal development, maturation and maintenance of the eye and the potential therapeutics thereof.

## Cranial Neural Crest Migration Meets Optic Cup Formation

**FIGURE 1:**
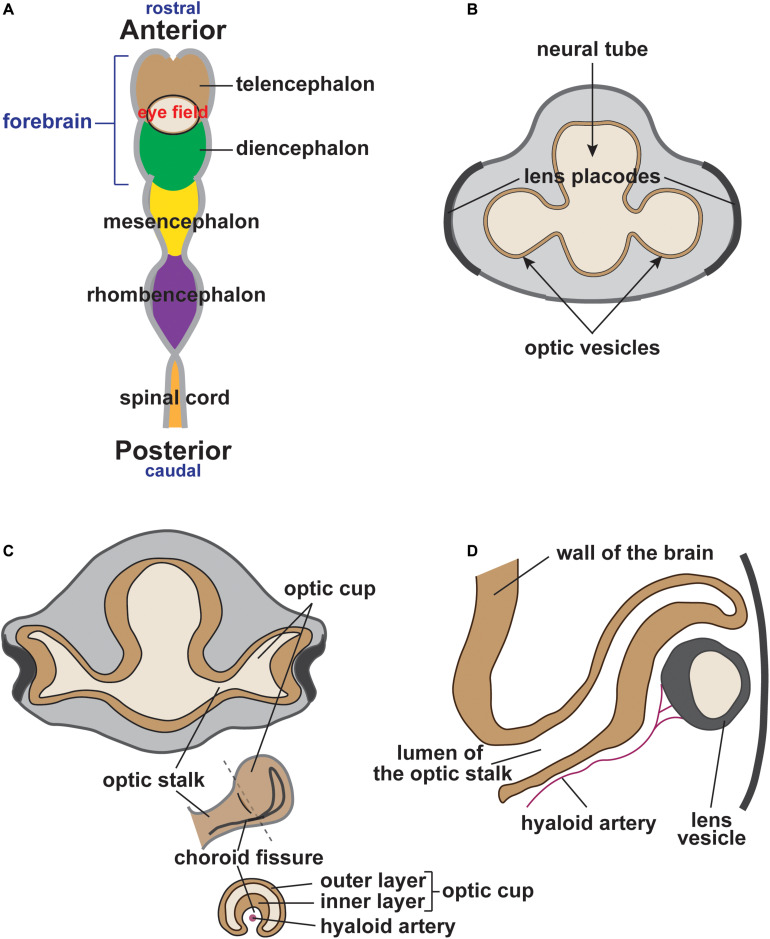
Vertebrate eye development (human eye). **(A)** During early vertebrate development, the eye field is established at the boundary between the telencephalon (brown) and the diencephalon (green). **(B)** Optic vesicles bilaterally protrude from either side of the forebrain approaching the thickened surface ectoderm (lens placodes). **(C)** The interaction between the optic vesicle and the lens placode of the surface ectoderm results in optic vesicle invagination, optic cup formation and lens placode evagination (lens pit). Simultaneously, the optic fissure is formed along the inferonasal aspect of the optic cup, which surrounds the hyaloid artery. **(D)** Continued evagination of surface ectoderm leads to the formation of an independent lens vesicle.

**FIGURE 2:**
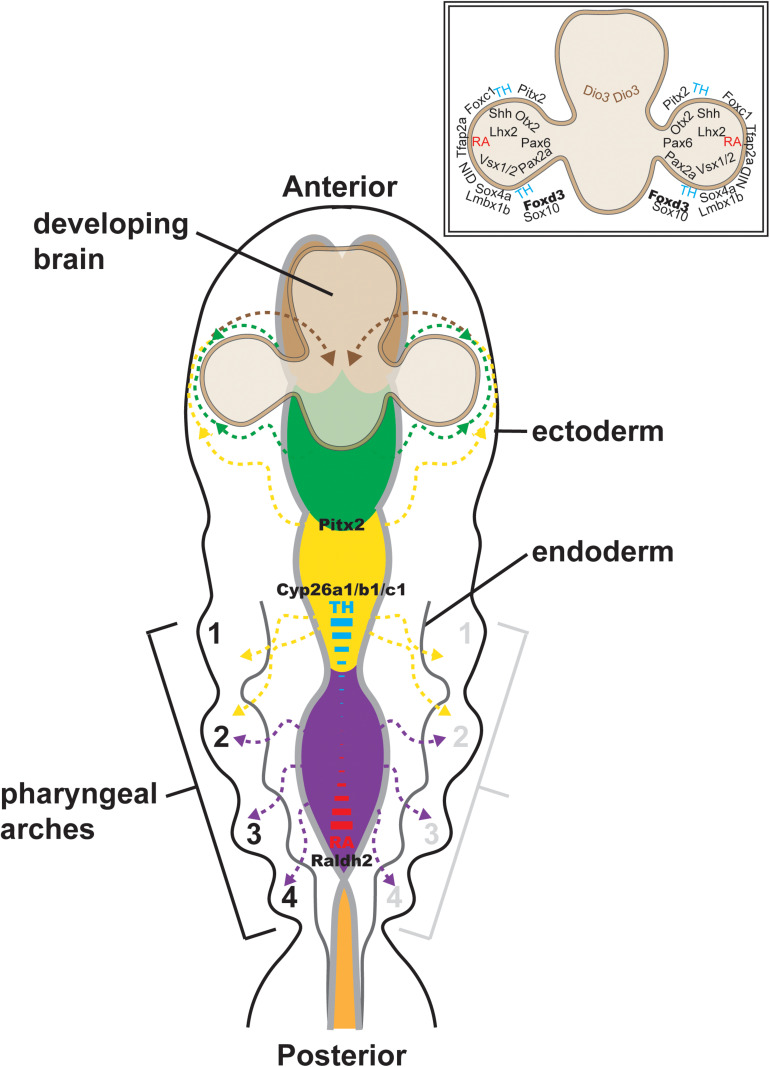
Cranial neural crest migration into the head. Time-lapse imaging of NC cell migration in zebrafish has shown that cranial NC cells from the diencephalon (green dashed lines and arrows) and anterior mesencephalon (anterior yellow dashed lines and arrows) travel either dorsal or ventral to the optic cup to establish the POM. A subpopulation of these cells migrates anteriorly into the frontonasal process (brown dashed lines and arrows). NC cells from the posterior mesencephalon (posterior yellow dashed lines and arrows) and rhombencephalon (purple dashed lines and arrows) migrate toward the pharyngeal arches on the ventral side of the embryo. Some of the factors implicated in the regulation of NC migration (Pax6, Pitx2, Foxd3, Sox10, Foxc1, etc.) are shown (inset, upper right). The signaling gradient that maintains the integrity of cranial NC cell migration [i.e., thyroid hormone (TH; blue) and retinoic acid (RA; red)] into the posterior pharyngeal arches is also shown.

Central to understanding eye development are the concurrent events intertwining cranial NC development and optic cup morphogenesis. During late gastrulation, the developing eye is established as a single eye field located in the medial anterior neural plate at the boundary between the telencephalon and diencephalon (reviewed in Miesfeld and Brown, 2019; Figure 1A). Concurrent with the delamination of NC cells from the neural plate border, the eye field undergoes bilateral bifurcation to cause the evagination of the left and right optic vesicles (Figure 1B). Upon contact with the overlying surface ectoderm, the optic vesicles invaginate to form bilayer optic cups (Figure 1C). The interaction between the optic vesicle and the lens placode of the surface ectoderm induces lens vesicle evagination and establishes early ocular anterior segment formation (Figures 1C,D). During this process, the optic fissure is formed along the inferonasal aspect of the cup as it envelops the hyaloid vasculature (Figure 1C). Meanwhile, cranial NC cells that have undergone EMT migrate into the developing head (Figure 2). Cells from the diencephalon and anterior mesencephalon divide into two waves that move either dorsal or ventral to the optic cup. A subpopulation of these cells remains within the POM, while additional cells travel anteriorly into the frontonasal process. NC cells from the posterior mesencephalon and rhombencephalon take a more direct route toward the pharyngeal arches on the ventral side of the embryo.

In zebrafish, there is interplay between thyroid hormone and retinoic acid in regulating the different streams of cranial NC (reviewed in Williams and Bohnsack, 2019; Figure 2). NC cell migration into the posterior pharyngeal arches requires a low level of thyroid hormone, but a high level of retinoic acid. This requirement corresponds with the high levels of retinoic acid centered within the posterior rhombencephalon by the spatial expression of retinoic acid synthesis (retinaldehyde dehydrogenase 2; Raldh2) and degradation (cytochrome P450 family 26; Cyp26a1, Cyp26b1, and Cyp26c1) enzymes. In contrast, NC cell migration into the first pharyngeal arch, which most prominently gives rise to the maxilla and mandible bones, requires high thyroid hormone and low retinoic acid levels. The reciprocal effects of thyroid hormone and retinoic acid may be mediated by their shared retinoid X receptor (RXR). In anterior cranial NC cells, thyroid hormone is required for initiation of the migratory stream ventral to the eye, but then localized degradation by iodothyronine deiodinase 3 (Dio3) is necessary for completion of their migratory arc from the POM into the frontonasal process. On the other hand, the dorsal NC migratory stream requires a high level of thyroid hormone for both the initiation and completion of migration. In addition to its location at the posterior rhombencephalon, retinoic acid is also concentrated at the dorsal and ventral optic cup. This high level of retinoic acid is required for the proper migration of NC streams both dorsal and ventral to the eye as well as the migration of the POM NC population (Bohnsack and Kahana, 2013). The transcription factor paired like homeodomain 2 (Pitx2), well known for its association with Axenfeld-Rieger syndrome (ARS) (discussed below), also regulates these early stages of cranial NC migration. In zebrafish, morpholino oligonucleotide knockdown of Pitx2 expression inhibits migration and induces the apoptosis of NC cells derived from the mesencephalon, thereby resulting in decreased NC populations within the first pharyngeal arch and the ventral POM (Bohnsack et al., 2012; Ji et al., 2016).

Following migration, the NC cells within the POM surround the optic vesicle and play an important but ill-defined role in establishing the optic stalk and cup (Figure 2). Indeed, Pitx2 gene knockout mice show severe eye phenotypes, including anophthalmia (lack of eye), microphthalmia (small disorganized eye) and optic nerve defects, such as eyes that attach directly to the ventral hypothalamus (Evans and Gage, 2005). Importantly, NC-targeted Pitx2 conditional gene knockouts show a similar phenotype (Liu and Semina, 2012), indicating that these eye abnormalities reflect the cell non-autonomous effects of Pitx2 in NC cells. However, a direct connection between Pitx2 and known regulators of optic stalk and cup development, such as sonic hedgehog (Shh), paired box protein 2 (Pax2), Pax6, retinal homeobox protein (Rax), visual system homeobox 1 (Vsx1), Vsx2, orthodenticle homeobox 2 (Otx2) and LIM homeobox protein 2 (Lhx2), has yet to be established. Further, in zebrafish, loss of NC cells within the POM through genetic targeting of the NC specifiers, transcription factor AP-2 alpha (Tfap2a) and forkhead box D3 (Foxd3), disrupts optic cup invagination and optic fissure formation (Wang et al., 2011). NC cell production of the extracellular matrix protein, Nidogen (Nid), contributes to basement membrane formation around the optic vesicle and is critical for retinal pigment epithelial cell movements during cup invagination (Bryan et al., 2020). In addition, NC cells help to establish patterning of the optic cup, which is critical for optic fissure closure. The optic fissure extends from the optic stalk to the distal edge of the optic cup and transmits the transient hyaloid vasculature that nourishes the developing lens and retina. Closure of the optic fissure is a complex process that has not been fully elucidated at the molecular level, and the failure of fissure closure results in optic nerve, chorioretinal and iris colobomas. Dorsal-ventral patterning of the optic cup by a similar set of transcription factors important for optic stalk and cup development is critical for setting up fissure closure. Mutant animal models of these retinal genes are characterized by anophthalmia, microphthalmia, and coloboma. Similarly, knockdown of the NC-specific transcription factor, SRY-box transcription factor 4a (Sox4a), also causes coloboma, as decreased Sox4 gene expression results in the mislocalization of Pax2a, Vsx1, and Vsx2 within the retina (Wen et al., 2015). Thus, early eye morphogenesis and cranial NC development intersect at multiple points, with important crosstalk between the tissues that regulate optic cup formation and direct NC migration.

## Anterior Segment Development: Emergence of the Ocular Neural Crest

**FIGURE 3:**
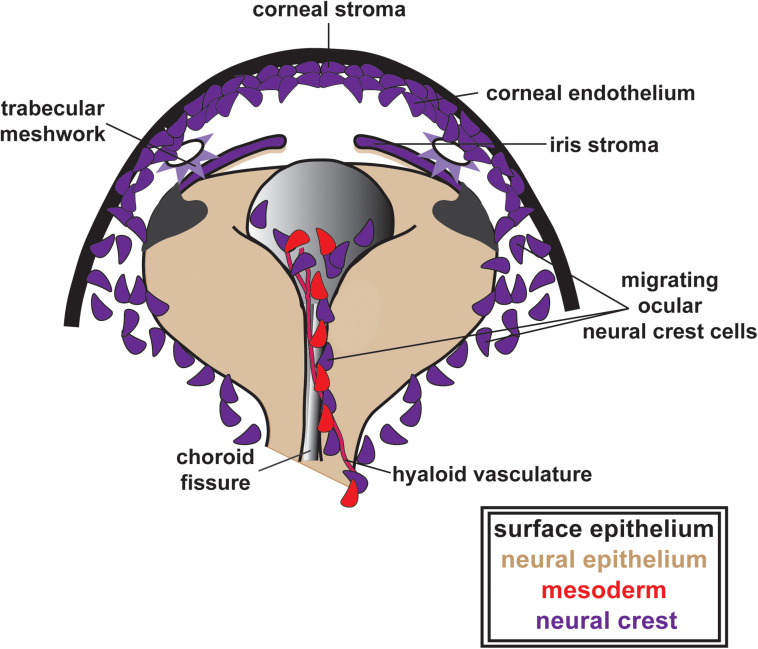
Ocular neural crest migration and establishment of the ocular anterior segment. POM NC cells (purple), along with mesoderm cells (red), migrate adjacent to the hyaloid vasculature within the optic fissure and between the surface ectoderm and the optic cup contributing to the corneal stroma and endothelium, the iris stroma and the trabecular meshwork. In this figure, these structures are all indicated in purple to illustrate their ocular NC derivation. Notably, as previously discussed in the text above, the patterns of ocular NC cell migration vary between species (three distinct waves of NC cells in humans, two NC cell waves in mice and a single continuous migratory wave in chick). However, for simplicity, these patterns are not depicted here.

Following optic cup morphogenesis, including the establishment of the optic fissure and anterior segment, NC cells within the POM begin migration into the eye (Figure 3). The patterns of migration vary between species. Classic anatomic studies in human embryos suggest three distinct waves of NC cells that contribute first to the corneal stroma and endothelium, second to the iris stroma and third to the trabecular meshwork (Williams and Bohnsack, 2015; Miesfeld and Brown, 2019). In contrast, there are two NC cell waves in mice and a single continuous migratory mesenchymal wave in chick (reviewed in Miesfeld and Brown, 2019). Fate mapping studies in mice have shown that both NC and mesoderm contribute to the corneal stroma and corneal endothelium, while in chick, both of these corneal structures as well as the iris stroma are NC derived (Gage et al., 2005). Time-lapse imaging of pre- and post-migratory NC cells in zebrafish embryos has shown at least three waves, including two distinct Sox10*-*positive and one Foxd3-positive cell populations (Eason et al., 2017; Takamiya et al., 2020). Sox10-positive cells migrate early after optic cup formation and preliminary fate mapping studies show minimal contributions to the adult eye (unpublished data). Foxd3-positive NC cells migrate into the anterior segment via two pathways: (1) adjacent to the hyaloid vasculature within the optic fissure and (2) between the surface ectoderm and the optic cup (Mork and Crump, 2015; Williams and Bohnsack, 2017; Van Der Meulen et al., 2020; Figure 3). Foxd3-positive cells have a continued presence within the corneal endothelium and iris in zebrafish larvae and young juveniles. However, Foxd3 expression is lost by adulthood and fate mapping of this population of cells has yet to be accomplished.

With the loss of early markers, such as Sox10 and Crestin, NC cells adopt the expression of other transcription factors, namely Pitx2, Foxc1, Lmx1b, and Eya2 (Van Der Meulen et al., 2020). Further, signaling molecules, such as retinoic acid, and interactions between NC cells and the adjacent ocular tissues have continued effects on the ocular migration and end differentiation of NC cells. To date, much of the knowledge regarding NC cell contributions to the anterior segment of the eye derives from clinical and basic science studies focused on the pathogenesis of congenital eye diseases.

## Ocular Neural Crest Derivatives: Lessons From Rare Ocular Diseases

### Axenfeld-Rieger Syndrome (OMIM 180500)

**FIGURE 4:**
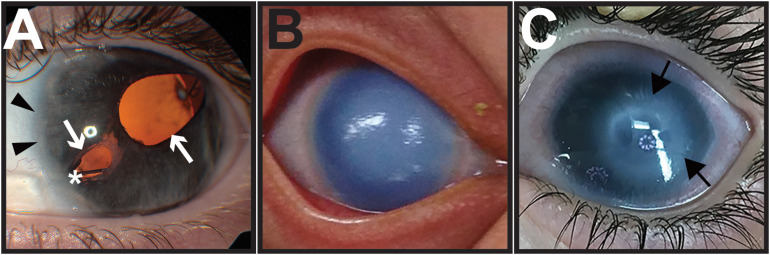
Congenital eye diseases associated with neural crest defects in the anterior segment. **(A)** Axenfeld-Rieger syndrome is characterized by anteriorization of Schwalbe’s line (posterior embryotoxon, black arrowheads) and iris hypoplasia, causing corectopia and pseudopolycoria (white arrows). Over half of affected individuals develop glaucoma, and many require placement of a glaucoma drainage device (asterisk) to control intraocular pressure. **(B)** Primary congenital glaucoma is due to developmental abnormalities in the trabecular meshwork and aqueous outflow tracts. As a result, elevated intraocular pressures in infants cause corneal edema and buphthalmos (increased eye size). **(C)** Peters anomaly shows central corneal opacification (black arrows) reflecting the abnormal separation of the lens vesicle from the surface ectoderm.

Axenfeld-Rieger syndrome is characterized by congenital malformations affecting NC-derived craniofacial and ocular structures. Most prominent are the ocular abnormalities that combine Axenfeld anomaly (posterior embryotoxon) and Rieger anomaly (iris hypoplasia), resulting in corectopia (irregular pupil), and pseudopolycoria (multiple pupils) (Figure 4A). Over 50% of affected individuals have glaucoma, which due to iris and trabecular meshwork abnormalities, often requires multiple surgeries to preserve vision (Zepeda et al., 2020). In addition, individuals with ARS may have a distinctive craniofacial structure that includes telecanthus, maxillary hypoplasia, and broad flat nasal bridge, teeth abnormalities, such as oligodontia and microdontia, and congenital heart abnormalities.

ARS shows genetic heterogeneity, and autosomal dominant mutations in Pitx2 and Foxc1 genes account for up to 70% of cases (Reis et al., 2012; Seifi et al., 2017; Zamora and Salini, 2020). Based on the clinical correlation with ARS, these two homeobox transcription factors play critical roles in craniofacial and ocular NC development. In mice and zebrafish embryos, Pitx2 is expressed in NC cells soon after delamination, and as discussed above, this factor regulates migration into the pharyngeal arches and POM (Chawla et al., 2016). In zebrafish, Pitx2 knockdown causes decreased NC cell migration within the pharyngeal arches, and this effect accounts for the maxillary hypoplasia and cardiac outflow tract and teeth anomalies seen in human disease. Further, in mice, complete knockout of Pitx2 results in severe heart defects and embryonic lethality that precludes full craniofacial and ocular analysis (Kitamura et al., 1999; Evans and Gage, 2005). In contrast, mice heterozygous for the Pitx2 null allele and those in which the Pitx2 knockout was limited to NC cells were viable and displayed corneal, iris, and scleral defects similar to ARS in humans (Chen and Gage, 2016). Retinoic acid is a known regulator of Pitx2 expression (Bohnsack et al., 2012; Williams and Bohnsack, 2015; Chawla et al., 2016; Hebert et al., 2017; Draut et al., 2019; Williams and Bohnsack, 2019), but has differential effects depending on embryonic stage. During the early stages, retinoic acid decreases Pitx2 expression within NC cells migrating toward the pharyngeal arches. However, during later NC cell migration, retinoic acid increases Pitx2 expression within the periocular region in both mice and zebrafish (Gage et al., 2008; Duester, 2009; Kumar and Duester, 2010; Zacharias and Gage, 2010; Bohnsack et al., 2012). Additional studies in mice have shown that paracrine retinoic acid signaling in the POM, mediated via nuclear retinoic acid receptors RXR alpha, RAR beta and RAR gamma, not only regulates the remodeling of the POM, but also controls the expression of both Foxc1 and Pitx2 (Matt et al., 2005; reviewed in Williams and Bohnsack, 2019). Moreover, defects in retinoic acid signaling in NC cells are sufficient to completely reshape eye development and mediate patterning of the optic vesicle (Matt et al., 2008). Lymphoid enhancer-binding factor 1 (Lef-1) and β-catenin bind the Pitx2 gene promotor in mice, indicating a role for the canonical Wnt signaling pathway in regulating Pitx2 expression. Notably, Pitx2 regulates the Wnt antagonist, Dkk and thus forms a regulatory feedback loop within NC cells.

An additional target of Pitx2 is the LIM-homeodomain transcription factor, Lmx1b, which is initially expressed in POM NC cells and then subsequently in the iris, cornea, trabecular meshwork, and hyaloid vasculature. Lmx1b plays a role in NC cell migration and survival, such that homozygous mutant mice have microphthalmia with iris and ciliary body hypoplasia (Pressman et al., 2000). Autosomal dominant Lmx1b gene mutations cause Nail-Patella Syndrome (OMIM 161200), in which affected individuals are at risk for open angle glaucoma, but do not exhibit congenital eye abnormalities (Vollrath et al., 1998). This suggests that heterozygosity in the Lmx1b gene is adequate for ocular NC cell development, but not for postnatal maintenance of the structure and function of the trabecular meshwork. In mice, Pitx2 also regulates Tfap2b, which is expressed in NC cells in the POM and regulates anterior chamber development via a Lmbx1b-independent pathway (Chen and Gage, 2016). Human Tfap2b gene mutations are associated with autosomal dominant Char syndrome (OMIM 169100), which is characterized by craniofacial abnormalities, but not eye defects. However, NC-specific Tfap2b knockout mice had closed iridocorneal angles due to abnormal iris, cornea, trabecular meshwork, and ciliary body development (Martino et al., 2016). In addition, Tfap2b regulates Zp4, Col8a2, and N-Cadherin in the corneal endothelium (Martino et al., 2016).

Fewer studies have focused on Foxc1 and NC cell development. In mice and zebrafish, genetic knockout or morpholino knockdown of Foxc1 are also embryonic lethal, while heterozygous animals show ocular abnormalities consistent with ARS (Smith et al., 2000). Foxc1 mediates the development of the corneal endothelium and maintains the avascular nature of the cornea, which is necessary for the transparency of this tissue and normal vision (Seo et al., 2012; Koo and Kume, 2013). Additionally, Foxc2 mutations hamper the specification of corneal epithelial cells and leads to ectopic corneal neovascularization and corneal conjunctivalization in mice (Seo et al., 2017), suggesting that both Foxc1 and Foxc2 collectively ensure precise ocular surface development. Pitx2 and Foxc1 colocalize within cells, and each of their genes affects the activity of the other. Indeed, in mice, Pitx2 interacts with the activation domain and negatively regulates the activity of Foxc1 (Berry et al., 2006). In contrast, decreased levels of Foxc1 and Foxc2 activity within NC cells have been associated with significant declines in Pitx2 expression and a more severe phenotype, including microphthalmia, corneal opacification, and eyelid fusion in these animals (Seo et al., 2017). In animal models, Eya2, Fgf19, Foxo1a, and Galnt4 have also been identified as downstream targets of Foxc1 in NC cells. However, unlike the Pitx2 targets, the clinical significance of these genes is not understood, as none of these genes are associated with cranial NC or eye abnormalities.

Taken together, the regulation and interaction between Pitx2 and Foxc1 in the NC are essential for craniofacial and ocular (corneal) development, and mutations in either of their genes manifests as the rare congenital disease, ARS. Nonetheless, additional studies are required to identify further downstream targets of Pitx2 and Foxc1 and determine their roles in anterior segment development.

### Primary Congenital Glaucoma (OMIM 231300)

Primary congenital glaucoma (PCG) is due to abnormal formation of the trabecular meshwork and aqueous outflow tracts resulting in increased intraocular pressure. PCG presents between birth and 2 years of age, but most commonly in the first 6 months with the classic triad of photophobia (light sensitivity), epiphora (tearing), and blepharospasm (frequent blinking/eye closure) (Figure 4B). These symptoms are due to corneal edema and Haab’s striae (breaks in Descemets layer), which are a result of elevated intraocular pressure. Further, PCG is characterized by buphthalmos (enlarged eye) with subsequent axial lengthening and myopic shift as well as glaucomatous optic neuropathy. Children with PCG require urgent surgery to lower the intraocular pressure and preserve vision.

Primary congenital glaucoma is most commonly associated with autosomal recessive mutations in the Cyp1b1 gene (Li et al., 2011). Independent of the retinoic acid catalysis enzymes Raldh2, Raldh3, and Raldh4, Cyp1b1 regulates the two-step conversion of vitamin A first to retinaldehyde and then to retinoic acid (Chambers et al., 2007). However, endogenous *in vivo* targets of Cyp1b1 have yet to be verified. In zebrafish embryos, Cyp1b1 is expressed within the retina in the inferior optic fissure and lesser-known superior optic fissure in correlation with optic fissure patency (Chambers et al., 2007; Williams and Bohnsack, 2017; Hocking et al., 2018). Indeed, the overexpression of Cyp1b1 prevents fissure closure, resulting in colobomas in zebrafish (Williams and Bohnsack, 2017), and a handful of case reports of superior coloboma have been associated with Cyp1b1 gene mutations (Hocking et al., 2018). In contrast, knockdown of Cyp1b1 causes premature closure and inhibits later NC migration through the inferior optic fissure, and this affect is independent of retinoic acid (Williams and Bohnsack, 2017). The sequence of NC differentiation in the anterior segment is first cornea, then iris and subsequently aqueous outflow tracts. Thus, one hypothesis is that the lack of later NC migration due to decreased Cyp1b1 specifically causes abnormalities in aqueous outflow, resulting in PCG. Additional studies in mice have demonstrated expression of Cyp1b1 in the trabecular meshwork (Zhao et al., 2015), and heterozygosity of Cyp1b1 gene mutations has been associated with the presentation of juvenile onset glaucoma after the age of 2 years. This finding suggests that Cyp1b1 plays a role in trabecular meshwork formation and maintenance. However, the molecular targets and cellular functions of Cyp1b1 within this tissue are unknown.

### Peters Anomaly (OMIM 604229)

Peters anomaly (PA) is a congenital eye disease characterized by central corneal opacification due to the abnormal separation of the lens from the overlying surface ectoderm. The resulting abnormal migration and differentiation of ocular NC cells leads to iris-corneal adhesions and absence of corneal endothelium and Descemets membrane (Figure 4C). In severe cases, the lens is also adhered to the cornea, which can cause corneal staphylomas (Alallah et al., 2020; Chang et al., 2020; Katz et al., 2020; Kletke et al., 2020; Samara and Eldaya, 2020). In addition, cataract, microphthalmia, and aniridia (iris absence or hypoplasia) are commonly associated with PA. Glaucoma complications due to a closed angle configuration and abnormal trabecular meshwork and aqueous outflow tract formation are observed in approximately two-thirds of cases and often requires multiple surgeries to obtain intraocular pressure control (Ozeki et al., 2000; Gould and John, 2002; Sowden, 2007; Harissi-Dagher and Colby, 2008; Dolezal et al., 2019). Although most cases are isolated to the eye, systemic findings can include craniofacial anomalies, congenital heart defects, and developmental delay (Dolezal et al., 2019).

Genetic mutations in or chromosomal deletions involving known genes that regulate anterior segment development have also been associated with PA. While Pitx2 and Foxc1 are typically affiliated with ARS and Cyp1b1 is associated with PCG, mutations in these genes have been linked to rare cases of PA. Notably, the underlying molecular pathogenesis of the mutations in these genes that lead to PA versus their more commonly associated congenital diseases is not well understood (Honkanen et al., 2003; Weisschuh et al., 2008; Berker et al., 2009; Arikawa et al., 2010; Gage et al., 2014; Hassed et al., 2017).

Pax6 gene mutations have also been linked to PA. Notably, mutations in Pax6 are more typically associated with aniridia, a panocular congenital disease characterized by foveal hypoplasia, optic nerve hypoplasia, limbal stem cell deficiency, and varying degrees of iris hypoplasia (reviewed in Lim et al., 2017; Sannan et al., 2017; Syrimis et al., 2018; Wawrocka and Krawczynski, 2018; Lima Cunha et al., 2019; Lee et al., 2020; Tripathy and Salini, 2020). However, phenotypic variation of the same Pax6 gene mutation has shown both aniridia and PA within one family (Wang et al., 2018), and PA has been associated with aniridia in more than 10% of cases (Dolezal et al., 2019).

Animal models have shown that Pax6 targets essential extracellular signaling molecules that control multiple steps in eye morphogenesis (Cvekl and Callaerts, 2017). Pax6 is initially expressed in the optic pit and subsequently in the optic vesicle, optic stalk and overlying surface ectoderm (Enwright and Grainger, 2000; Grocott et al., 2011; Cvekl and Callaerts, 2017; Takamiya et al., 2020). Pax6 is important for optic stalk formation and retinal differentiation, which correlates with optic nerve and foveal hypoplasia observed with human Pax6 gene mutations. Further, during cup morphogenesis, Pax6 expression is primarily restricted to the surface ectoderm and the distal end of the optic cup. This expression underpins formation of the lens placode and subsequent detachment of the lens from the cornea (Takamiya et al., 2020) and helps to explain the relationship between Pax6 mutations and PA. Notably, Pax6 gene mutations and deletions yield phenotypic differences between humans and animal models. While Pax6 gene mutations in humans are most commonly associated with aniridia (Lee et al., 2020; Tripathy and Salini, 2020), in mice and zebrafish, microphthalmia and severe anterior segment dysgenesis are observed (Takamiya et al., 2015; Yasue et al., 2017; Grant et al., 2020). Although not well understood, these phenotypic differences among species demonstrate the multiple essential roles of Pax6 in eye development.

Mutations within Pitx2, Foxc1, Cyp1b1, and Pax6 only account for a small percentage of cases of PA. Further clinical genetic analyses of PA cases have revealed the importance of additional transcription factors, including Pitx3 and Foxe3 for ocular development. Clinical and histological analyses revealed adhesions between either the iris or lens and the cornea, with the anterior dislocation of Schwalbe’s line and cataracts in families with a 17-bp insertion mutation in the Pitx3 gene (Summers et al., 2008). In addition, targeted sequencing has revealed other known and novel mutations in Pitx3 in PA patients (Zazo Seco et al., 2018). In mice, Pitx3 is expressed in the lens, and homozygous mouse mutants with lenticular expression of truncated Pitx3 presented with microphthalmia and aphakia (Wada et al., 2014). Further, a homozygous guanine insertion (c.415_416insG) mutation in the Pitx3 gene in mice resulted in an eyeless phenotype characterized by closed lids, corneal thickening, and corneolenticular adhesion (Rosemann et al., 2010). In addition, Pitx3 positively regulates Foxe3 expression in the anterior lens epithelium, and although these two genes do not interact directly, Pitx3 knockdown in mice and zebrafish eliminates Foxe3 expression. While Foxe3 mutations are typically associated with congenital aphakia (absence of lens), there have been reports of PA (Garcia-Montalvo et al., 2014). In mice, Foxe3 mutations result in corneolenticular adhesions, abnormally shaped lens fiber cell nuclei, chorioretinal folds, missing corneal endothelium, disarrayed corneal stroma, and reduced corneal epithelium thickness (Medina-Martinez et al., 2005). Further, a significant reduction in lens epithelial cell proliferation has been observed, and the differentiation of these cells occurred appreciably earlier than that in wild-type mice (Medina-Martinez et al., 2005).

In addition to genetic alterations, *in utero* exposure to environmental toxins, such as alcohol, can disrupt anterior segment development and lead to PA. Fetal alcohol spectrum disorders encompass a wide range of behavioral and physical defects that result from alcohol consumption during pregnancy. Fetal alcohol syndrome (FAS) is the most severe form, and this disease is characterized by neurologic and cognitive disabilities and typical craniofacial abnormalities, such as a thinned vermillion, shortened palpebral fissures, a cleft palate, and/or cleft lip (Vorgias and Bernstein, 2020). In addition, the eyes and vision are often affected, most commonly showing neuroepithelial-derived optic cup abnormalities, such as microphthalmia and optic nerve hypoplasia. In rare cases, FAS is also associated with PA, suggesting that the steps in eye development are sensitive to alcohol exposure. Interestingly, despite the almost universal craniofacial features in FAS, the NC-derived anterior segment is less commonly affected (Brennan and Giles, 2014). This finding suggests molecular distinctions between different NC cell populations, i.e., cranial NC cells that give rise to craniofacial structures versus those that give rise to the ocular structures. Interestingly, compared to Sox10-expressing NC cells fated to craniofacial structures, the survival and migration of ocular Foxd3-positive NC cells were less sensitive to the effects of alcohol. To some extent, this difference was mediated by a higher basal level of mitochondrial superoxide dismutase (Sod2) in the anterior segment of the eye compared to that in the craniofacial NC, resulting in a greater capacity to activate oxidative stress in response to ethanol exposure in the eye (Eason et al., 2017). These results are consistent with the propensity for craniofacial defects versus the rarity of ocular anomalies in FAS. A recent study showed that miRNA-135a overexpression markedly decreased ethanol-induced apoptosis in NC cells in zebrafish embryos, and the microinjection of miRNA-135a mimics ameliorated growth retardation and craniofacial defects (Yuan et al., 2020). These results suggest that microRNAs play a role in NC-mediated ocular and craniofacial development.

Taken together, anterior segment development requires the coordinated movement of cells and tissues derived from the surface ectoderm, neural epithelium and NC. Genetic or toxic disruption of lens formation and subsequent NC cell migration and differentiation results in a common phenotype of PA. However, the identification of genetic mutations is lacking in a majority of cases, indicating that many other yet to be identified genes are important during this process. Additional studies are required not only to fully elucidate this disease pathogenesis but also to understand the molecular regulation of anterior segment development.

## Theories of Disease Mechanisms: Implications of Dosage and/or Functional Effects

Most studies on the NC have focused on characterizing the genes that coordinate the development of the cranial NC and its multiple derivatives. However, as the ocular NC appears to be the source of the tissues involved in rare congenital diseases, it is also important to understand how specific cellular defects and implicated genes ultimately lead to phenotypic abnormalities, as this information could considerably increase our understanding of the end tissue differentiation of this NC cell population and improve the management and prevention of ocular diseases. In particular, disorders of the ocular anterior segment, commonly referred to as anterior segment dysgenesis, result from dynamic interactions between embryological, genetic, and developmental factors with frequent phenotypic overlap and extensive mutations at more than one genetic locus, and, for the most part, the mechanisms by which these gene mutations result in disease are not precisely known, but often relate to dosage sensitivity or alterations in protein function.

### Dosage Effects

Alterations (either increased or decreased) in the levels of functional Pitx2 and Foxc1 cause disease. Without question, both proteins show dosage sensitivity, as minute deletions of either Foxc1 or Pitx2 lead to ARS by way of haploinsufficiency, and a previous study reported an ARS phenotype that may also result from the genetic duplication of Foxc1 (Berry et al., 2006). In addition, zebrafish carrying a heterozygous Foxc1 gene deletion mutation showed ocular defects from haploinsufficiency consistent with the ocular manifestations in their human counterparts (Ferre-Fernández et al., 2020). Similarly, an assessment of the requirements for Pitx2 showed that heterozygotes for either hypomorphic or null alleles in the Pitx2 gene have eye abnormalities consistent with ARS, indicating a Pitx2 dosage requirement for eye development in mice (Gage et al., 1999a,b). In zebrafish, morpholino knockdown of all known alternative Pitx2 proteins resulted in abnormal craniofacial and ocular development (Liu and Semina, 2012). Notably, hypermorphic alleles of the Pitx2 gene have been identified in rare cases of ARS (Priston et al., 2001), and a phenotype consistent with ARS was demonstrated in zebrafish following the microinjection of mRNA transcribed from plasmid DNA carrying a dominant negative gene mutation (K50E) in the human Pitx2 gene (Bohnsack et al., 2012). These data further reinforce the idea that Pitx2 levels must be strictly regulated for normal ocular development and function.

Notably, in vertebrates, Pax6 function is also hallmarked by gene dosage, and aniridia in humans is linked with mutations that lead to Pax6 haploinsufficiency. Indeed, the high and continuous expression of Pax6 in ectodermal tissues, such as the cornea, corneal epithelium, ciliary epithelium iris, and lens, is indispensable for the expression of structural genes (crystallins and cell adhesion molecules), signaling molecules affecting ocular NC migration (reviewed in Cvekl and Callaerts, 2017) and auxiliary transcription factors, including Six3, cellular musculoaponeurotic fibrosarcoma (c-Maf) and prospero homeobox 1 (Prox1) (Cvekl et al., 2004). Moreover, low transitory levels of Pax6 gene expression are necessary for the establishment of the aqueous outflow system (i.e., trabecular meshwork differentiation) and the generation of the corneal endothelium (Baulmann et al., 2002).

### Functional Effects

Missense mutations in the genes implicated in ocular diseases result in alterations in protein functions. For example, although Pitx2 haploinsufficiency has been demonstrated as a mechanism of ARS, a majority of the reported Pitx2 gene mutations indicate reduced or abolished protein function resulting from defective DNA binding, decreased target gene induction or both (Cox et al., 2002; Tumer and Bach-Holm, 2009). Similarly, biochemical analyses of human ARS-causing mutations revealed severe ARS mutations that completely disrupted DNA binding activity and significantly decreased the transactivation activity of Pitx2 (Kozlowski and Walter, 2000; Priston et al., 2001; Espinoza et al., 2002). These results showed that variations in Pitx2 activity, and thereby function, underlie the range of phenotypes observed in disorders affecting the anterior segment in ocular development. In addition, missense mutations of Foxc1 also show variable effects on its nuclear localization, DNA-binding ability and transactivation activity (Kozlowski and Walter, 2000; Priston et al., 2001; Espinoza et al., 2002; Seifi et al., 2017). The glaucomatous mutations of Cyp1b1 also reportedly decrease the stability, abundance, or catalytic activity of this enzyme (Jansson et al., 2001; Chavarria-Soley et al., 2008; Choudhary et al., 2008; Campos-Mollo et al., 2009; Lopez-Garrido et al., 2010, 2013; Medina-Trillo et al., 2016). Notably, recent studies have demonstrated the significance of Cyp1b1 for the establishment and functional viability of the trabecular meshwork (Mookherjee et al., 2012; Zhao et al., 2013). Thus, mutations affecting the function of the Cyp1b1 gene may also disrupt the function of the trabecular meshwork, resulting in dysregulated intraocular pressure and glaucomatous optic nerve damage. Similarly, investigations of the functional consequences of dominant and recessive Foxe3 gene mutations have shown altered and loss-of-function transcriptional activity, respectively (Islam et al., 2015).

## Future Directions and Challenges

The normal formation, migration and differentiation of NC cells is key to the evolution of various tissues and systems. Studying the signaling pathways important for the development of the NC may increase progress in the management of degenerative diseases affecting NC-derived craniofacial and ocular structures. Notably, the extensive migration and differentiation of NC cells into diverse cell types is reminiscent of the migration, invasion and proliferation of metastatic cancer cells and the multipotency of stem cells (Kerosuo et al., 2015; Maguire et al., 2015; Gallik et al., 2017), suggesting similar pathways and molecular mechanisms between these cell types. Additionally, the signals that are important during embryogenesis have recently been highlighted as key factors in the maintenance of adult tissues, and new therapeutic targets of adulthood diseases may be discovered through enhancements or modifications of these pathways. Although much is known on the level of the cranial NC, inadequate knowledge of a NC-derived stem cell population with regenerative properties to reestablish the structures in the anterior eye (cornea, iris, lens, or aqueous tract) is a notable therapeutic challenge. Recent studies have provided information concerning a NC-derived limbal stem cell niche that holds promise for regenerative therapies (Rama et al., 2010; Utheim et al., 2013; Sasamoto et al., 2018; Yazdanpanah et al., 2019). However, many unresolved issues remain. Determining the molecular markers that differentiate cranial from ocular NC, establishing the differences between the NC cell populations that enter the eye via the ocular fissure versus those that migrate between the optic cup and surface ectoderm and characterizing differences between NC cells that form the iris versus the cornea are paramount to achieving a wholistic understanding of the various genes and pathways that will lead to novel and innovative treatment options.

## Author Contributions

All authors listed have made a substantial, direct and intellectual contribution to the work, and approved it for publication.

## Conflict of Interest

The authors declare that the research was conducted in the absence of any commercial or financial relationships that could be construed as a potential conflict of interest.
